# What Have We Learned (or Expect to) From Analysis of Murine Genetic Models Related to Substance Use Disorders?

**DOI:** 10.3389/fpsyt.2021.793961

**Published:** 2022-01-12

**Authors:** Gary Peltz, Yalun Tan

**Affiliations:** Department of Anesthesia, Pain and Perioperative Medicine, Stanford University School of Medicine, Stanford, CA, United States

**Keywords:** mouse genetic models, substance use disorder, neurobiologic basis, computational genetics, opiate addiction

## Abstract

The tremendous public health problem created by substance use disorders (**SUDs**) presents a major opportunity for mouse genetics. Inbred mouse strains exhibit substantial and heritable differences in their responses to drugs of abuse (**DOA**) and in many of the behaviors associated with susceptibility to SUD. Therefore, genetic discoveries emerging from analysis of murine genetic models can provide critically needed insight into the neurobiological effects of DOA, and they can reveal how genetic factors affect susceptibility drug addiction. There are already indications, emerging from our prior analyses of murine genetic models of responses related to SUDs that mouse genetic models of SUD can provide actionable information, which can lead to new approaches for alleviating SUDs. Lastly, we consider the features of murine genetic models that enable causative genetic factors to be successfully identified; and the methodologies that facilitate genetic discovery.

## Why Study Murine Genetic Models of SUD?

We believe that the relationship between murine models and human diseases (or biomedical traits) resembles that between a small Cessna airplane and a large 787 jet plane. You can learn most of what you need to know about the fundamental principles of aviation by studying the Cessna, but this will not enable you to pilot the 787. The 787 has many more capabilities, much more complex and computer-controlled systems, and multiple redundancies that are essential for its function than are contained within a Cessna. Nevertheless, you wouldn't be able to pilot a 787 without knowing the aviation principals that are learned by studying the Cessna. Similarly, studying the mouse has revealed the basic principles underlying many areas of human physiology and pathobiology. Within the neurobiology realm, many of the mechanisms and circuits utilized for learning, memory, cognition, and the effects that drugs have on these processes have been uncovered through analysis of mouse models. However, since laboratory mice function within a very limited behavioral domain and lack some of the neural pathways that regulate human behavior, many of the complex factors mediating human psychiatric diseases cannot be understood by analyzing rodent models. The aviation analogy is quite appropriate for SUDs. Rodent models are ideal for understanding DOA neurobiology and for providing information about how drug seeking behaviors are generated; but they provide a very poor substrate for investigating the impact of that socioeconomic and psychosocial factors have on triggering relapse. This is an important limitation since human drug addiction proceeds through a three-stage cycle whose intensity increases over time, and each stage results from DOA-induced changes in brain circuits (1–3). The first stage (binge/intoxication) is mediated by DOA-induced reward sensations in the brain. The second stage (withdrawal/negative affect) is characterized by an increased threshold for experiencing the reward sensation, and a withdrawal state develops when the DOA cannot be obtained. The third stage (preoccupation-relapse) is characterized by chronic relapse, which is triggered by environmental and emotional cues. Chronic DOA ingestion induces neurochemical changes that lessen the reward sensation that was experienced after DOA ingestion during the initial stage, which increases the stress and compulsivity associated with chronic drug addiction (2, 3). Mouse models are ideal for analyzing the first two stages of the addiction cycle, which are mediated by neurobiological changes that develop after acute (1st stage) or repeated (2nd stage) exposure to a DOA. In contrast, mice provide a less optimal model for analyzing 3rd stage phenomena, which involves responses to environmental triggers and far more complex DOA-induced changes that impact a wider range of neural circuits. Most current research and treatment efforts focus on the later stages of drug addiction (3), which are associated with drug craving and relapse in individuals with SUD of long duration. It could be more productive to increase the research effort devoted to developing prevention strategies, which target the early stage of drug addiction (4). To do this, we must develop a deeper understanding of DOA-induced changes at the synaptic level. In other words, to fly the jet plane (i.e., develop effective prevention or treatment methods for SUDs) we must use murine genetic models of SUD to understand the underlying principles of aviation (i.e., the mechanisms mediating SUDs).

Here, we examine what we have learned from our prior analyses of murine genetic models of responses related to SUD. First, we discuss a murine genetic model of a drug-induced toxicity to indicate the different types of genetic factors that can be identified. We then we review the genetic factors identified from our prior analyses of murine genetic models of opiate responses. Lastly, we consider the features of murine models that enable causative genetic factors to be successfully identified; and the methodologies that can facilitate genetic discovery.

## An Illustrative Example

Analysis of a murine genetic model of a drug-induced (haloperidol) CNS toxicity illustrates the potential outcomes that could emerge when evaluating murine genetic models of SUD because drug addiction (in many ways) is a toxicity caused by DOAs. Although haloperidol is an effective anti-psychotic agent, it causes a treatment-limiting side effect in most treated subjects, which is very debilitating Parkinsonian-like extrapyramidal symptoms. When we began our studies of haloperidol induced toxicity (HIT), genetic susceptibility factors for this toxicity were completely unknown. Therefore, we analyzed a murine genetic model of HIT where the inbred strains exhibited very large and reproducible differences in susceptibility to HIT. Our analysis revealed that susceptibility was quantitatively determined by two distinct genetic loci: one encoded a pharmacokinetic factor and the other a pharmacodynamic factor. The pharmacokinetic factor was allelic variation within a murine ABC-drug efflux transporter *(Abcb5)* that caused susceptible strains to have higher brain haloperidol levels; and a genetic association study in a haloperidol-treated human cohort identified human *ABCB5* alleles as susceptibility determinants for HIT (5). The pharmacodynamic susceptibility factor was allelic variation within pantetheinase genes (*Vnn1, Vnn3*) that impaired the biosynthesis of a protective metabolite (cysteamine) (6). While discovery of the murine pharmacokinetic factor led to the identification of a pharmacogenetic susceptibility factor for human HIT (5); characterization of the murine pharmacodynamic factor led to a potential new treatment (co-administration of a cysteamine metabolite) that could completely prevent haloperidol's treatment-limiting toxicity (6). Thus, analysis of a murine model generated information that produced a potential new method for preventing this toxicity.

## Murine SUD Models

**Figure 1:**
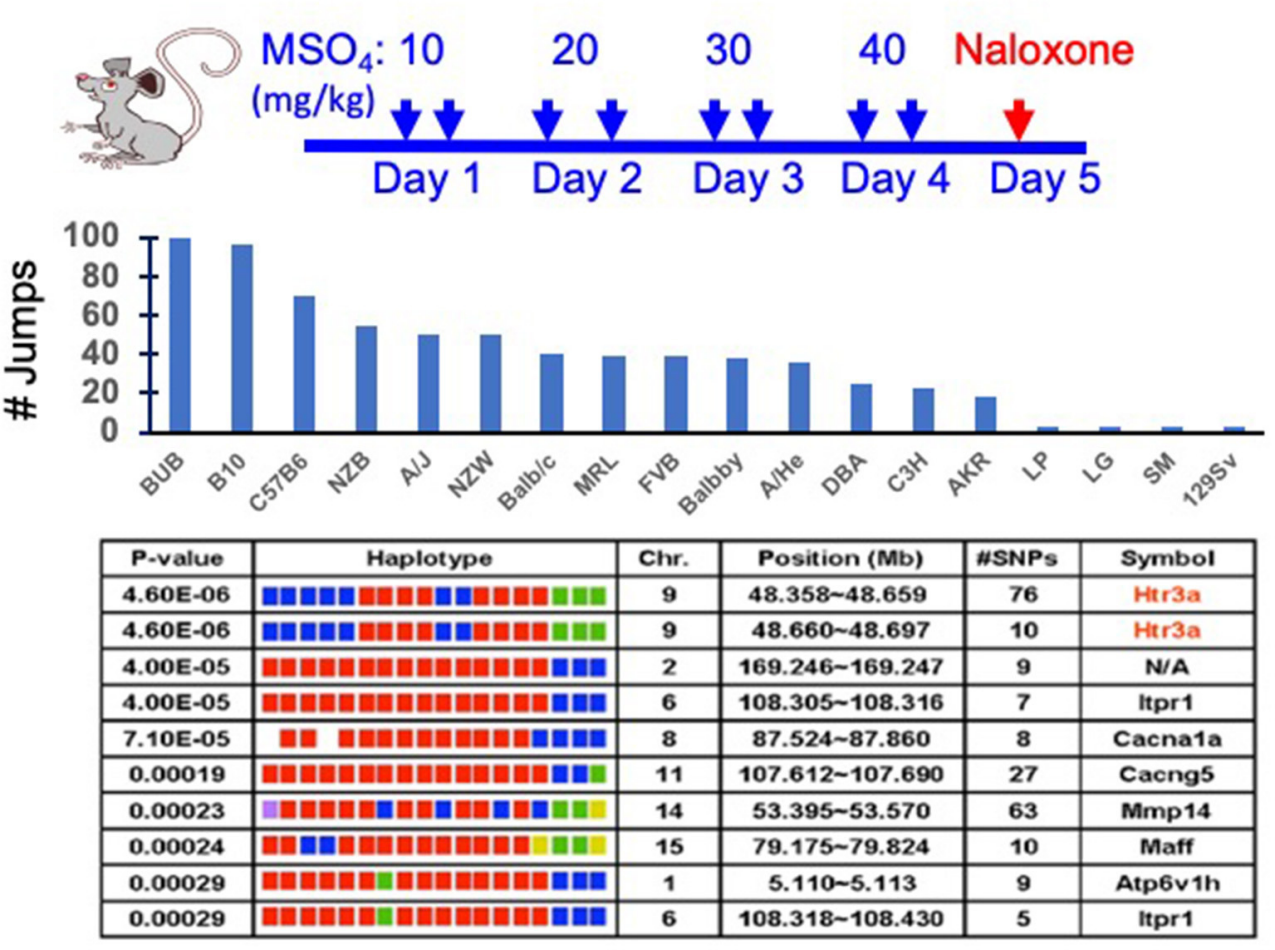
Analysis of a murine genetic model of naloxone precipitated opiate withdrawal (NPOW). **(Top)** Eighteen strains (eight mice per strain) were treated for four days with morphine to establish physical dependence. On the 5th day, the number of jumps made during the 15-min period after naloxone injection was measured to indicate the degree of opioid dependence. **(Middle)** The data represent the mean number of jumps for each indicated strain. **(Bottom)** The NPOW data (mean number of jumps for each strain) was analyzed by haplotype based computational genetic mapping. The 10 most strongly correlated haplotype blocks are shown. For each block, the chromosomal location, number of SNPs within a block and its gene symbol are listed. For each gene, the haplotypes are represented by a colored block, and the blocks are presented in the same rank order as the phenotypic data. Strains sharing the same haplotype have the same-colored block. The calculated *p*-value measures the probability that the strain groupings within a block would have the same degree of association with the phenotypic data by random chance. The genetic effect indicates the fraction of the inter-strain variance that is potentially attributable to the haplotype.

Like haloperidol, murine opiate response models hold great promise for genetic discovery. The inbred strains exhibit very large and heritable differences in their responses to opiates, which include the development of opioid analgesia, tolerance, dependence, and hyperalgesia (7–10). We provide a brief description of several rodent SUD models here, but more detailed information can be obtained from recent reviews covering rodent models for CPP (11), opioid (12, 13) and cocaine relapse (14), and opioid abstinence (15). The genetic models of SUD discussed here are ones where various responses are measured after DOAs are administered to panels of inbred mouse strains. For example, physical dependence is a key measure of addiction that is modeled by the jumping behavior that is displayed by opiate-dependent mice after administration of a potent opioid receptor antagonist (naloxone). This response is a highly heritable trait among inbred mouse strains (16) that is independent of differences in the method or duration of opiate administration (17, 18) (Figure 1). Of importance, naloxone-precipitated opiate withdrawal (NPOW) has also been used to quantify opioid dependence in human volunteers (19). In addition to their analgesic action, opioids also induce a paradoxical hypersensitivity to painful stimuli during opioid withdrawal (opiate-induced hyperalgesia, OIH); and there are large and heritable differences in the extent of OIH that develops among the inbred strains (7, 20). Drug seeking behavior is observed when abstaining addicts are confronted with environmental stimuli associated with their drug-taking behavior. Some features of the behavior of human opiate addicts can be modeled in mice using the morphine-induced conditioned place preference (mCPP) test (21–24). In the mCPP paradigm, morphine administration is paired with a particular spatial environment, and then a mouse's preference for this environment is measured to evaluate the rewarding properties of morphine. The OIH, NPOW and mCPP models measure phenomena in mice that are associated with the 2nd stage of the addiction cycle. OIH and withdrawal symptoms can serve as driving forces that promote relapse or escalation of drug intake. As such, the genetic factors identified from analysis of these models are ones that influence susceptibility to an SUD. Behavioral sensitization paradigms, which measure an increase in drug-induced behavior that gradually develops after a period of repeated DOA exposure, can also be used to study cross-sensitization amongst different DOA. Cross-sensitization studies using behavioral sensitization paradigms has identified the neural mechanisms and pathways that are shared by different types of DOA (25, 26). However, since these models are based on non-contingent (i.e., experimenter-initiated) drug administration, they lack face validity, which is the degree to which the model measures what it claims to. This aspect of addiction could be better studied using contingent models that assess the motivation for drug-taking or the reinstatement of drug-seeking behaviors (27).

Inbred strains also exhibit substantial and heritable differences in their cocaine responses, which include the extent of cocaine-induced locomotor activation (28, 29), cocaine self-administration (CSA) (30–33); and SUD risk-related behaviors that include impulsivity, and sensitivity to drug reward (33). Of the various addiction-related phenotypes studied in mice, the gold standard is operant self-administration (34, 35), where the subjects voluntarily and actively seek and consume drugs with rewarding properties. Rodents, like humans, experience the rewarding effects of a DOA, and they will engage in behaviors to procure them. To measure CSA, mice are fitted with an indwelling jugular catheter and placed in an operant conditioning box where they must depress a lever to trigger cocaine infusions. The rate of CSA reflects the reinforcing potential of cocaine (36). The substantial differences in CSA among the inbred strains (30–33) reflects their different propensities to abuse cocaine (37–39). There are obvious benefits from using a contingent model like CSA, since it more accurately recapitulates the drug-taking and drug-seeking behaviors of humans. The motivation (the reinforcing properties of the drug reward) as well as the specificity (drug vs. alternative reward) for drug-taking behaviors can also be evaluated in addition to measuring the quantity and frequency of drug administration (27, 40, 41). Thus, just as in the human population, inbred mouse strains exhibit substantial differences in their DOA responses; and characterization of the genetic basis for these differences will help us to understand the neurobiological effects of DOA and will enable us to understand how they generate addiction-related behaviors.

## Lessons Learned From Characterizing Murine Opiate Response Factors

As with HIT, multiple studies indicate that differences in the various types of opiate responses exhibited by inbred strains are determined by genetic factors that alter opiate pharmacokinetics and by pharmacodynamic factors that alter the host response to opiates. When a murine genetic model of opioid-induced hyperalgesia (OIH) was analyzed, we discovered that genetic variation within the *P-glycoprotein transporter* (*Abcb1b*) contributed to inter-strain differences in this opiate response (8). Analysis of the effect of pharmacologic inhibitors and of *Abcb1a/1b* knockout mice confirmed that P-glycoprotein function modulates narcotic-induced pain sensitization, as well as the tolerance and physical dependence that develops during opiate treatment. The brain morphine level correlated with the extent of OIH, which indicated a murine pharmacokinetic factor influenced multiple opiate pharmacodynamic responses by altering brain opiate levels. While pharmacokinetic factors are important, characterization of genetic factors affecting opiate pharmacodynamic responses are more likely to generate new approaches for preventing opiate addiction. For example, we analyzed another murine genetic model for OIH and identified the beta-2 adrenergic receptor (*Adrb2*) as a genetic locus contributing to the inter-strain response difference. This response was markedly diminished in *Adrb2* knockout mice and was reversed by administration of a commonly used *Adrb2* antagonist, which suggested a novel strategy for reducing OIH (7). We also found that genetic variation within genes encoding the *Netrin-1 receptor* (*Dcc*) (42) and *multi-PDZ-domain protein (Mpdz* that encodes MUPP1) (20) also contributed to inter-strain differences in the extent of tolerance, dependence and OIH that develops after repeated opiate exposure.

The latter two genetic findings indicate that opiate-induced changes at the synaptic level influence opiate responses. For example, *dcc* encodes a receptor for an axonal guidance protein (netrin-1) that plays a role in synaptic plasticity in the adult brain (43–46); and dcc itself plays a role in axonal differentiation and synaptogenesis in the developing brain (44, 46–48). Similarly, MUPP1 expression is localized to CNS synapses (49). Genetic variation within *Mpdz* has been associated with alcohol and sedative dependence in both mice and humans, which suggest that it may regulate responses to multiple DOA (50–52). MUPP1 may enhance the efficiency of neuronal signaling by bringing key intracellular signaling molecules into proximity with cell surface receptors (NMDA receptor) at the post-synaptic membrane (53). By this mechanism, NMDA receptor activation can trigger a MUPP1-facilitated cascade that leads to membrane insertion of AMPA receptor/channels, and persistent facilitation of glutamate signaling. This pathway may contribute to long-term potentiation (LTP) or alternative forms of enhanced AMPA receptor mediated activity (54). Pharmacological blockade of NMDA receptors and genetic deletion of NMDA receptor subunits has been shown to limit tolerance and OIH in mice and rats (55, 56); and the NR2B subunits of NMDA receptors mediate opiate tolerance (57, 58). The *dcc* and *Mpdz* findings also demonstrate that even when an identified causative genetic factor is not a pharmaceutic target, interacting proteins or proteins within an effected pathway may provide new therapeutic targets for SUD.

## Translation of a Mouse Genetic Discovery

Our most impactful discovery to date emerged from analysis of a murine genetic model that measured the naloxone-precipitated opiate withdrawal (NPOW) response after 4 days of morphine administration in 18 inbred strains (9). Allelic variation within the *Htr3a* gene encoding the 5HT_3A_R was most highly correlated with the severity of the NPOW response (Figure 1). Consistent with this result, *Htr3a* mRNA and protein expression was significantly reduced in a strain-specific manner after morphine administration. Moreover, administration of a selective 5HT_3A_R antagonist (ondansetron) reduced NPOW [and opioid-induced hyperalgesia (OIH)] in a dose-dependent fashion; and ondansetron co-administration with morphine impaired the mCPP response, which indicated that ondansetron eliminated the reinforcing effects of morphine (9). Thus, ondansetron also shows promise for preventing opiate dependence. The murine finding was tested in humans by measuring the effect of ondansetron on experimentally induced NPOW in healthy male volunteers. Ondansetron pre-treatment caused a 76% decrease (*p* = 0.03) in the NPOW in the volunteers, and it decreased all 11 of the measured manifestations of opiate withdrawal. *Since the ondansetron effect observed in mice translated to humans, it is likely of fundamental importance*. In a separate study (59), we demonstrated that another 5HT_3A_R antagonist (palonosetron) also prevented NPOW symptoms in normal human subjects and that a pretreatment that combined palonosetron with a commonly used antihistamine (hydroxyzine) caused a 95% reduction (*p* = 0.014) in withdrawal manifestations. The effect of the combination pretreatment was significant even when compared with that of palonosetron alone (*p* = 0.012) (59). *These results demonstrated that a 5HT*_3*A*_*R antagonist can be combined with another agent to further reduce opioid withdrawal severity*. Ondansetron is a widely used medication with a well-established safety record. After characterizing its pharmacokinetic properties in pregnant women and in their neonates (60), we are now performing a placebo-controlled clinical trial investigating whether a brief period of ondansetron treatment can prevent the development of opiate withdrawal symptoms in infants with prenatal opioid exposure (61, 62). This study, which has involved seven medical centers, currently represents the only attempt to develop a preventative treatment for a severe condition that effects the infants of mothers with SUD.

## Genetic Analysis Methods

Identification of the genetic factors responsible for DOA response differences among the inbred strains is an essential step for obtaining critically needed information about the neurobiological mechanisms underlying addiction. Only after a genetic factor is identified can the involved pathways be examined, which is required for identifying potential targets for new treatments for SUD. We have found that two inter-related features of a murine genetic model facilitate genetic discovery when genome wide association study (GWAS) methods are used for their analysis. (i) The DOA response must be measured across a large number (preferably > 15) of inbred strains. When a small number of strains are evaluated, the actual extent of the phenotypic variation present in the mouse population is under-estimated (63, 64). There are >450 available inbred strains (65); and usually only a few strains will exhibit an outlier phenotype for most responses. Unfortunately, the vast majority of murine GWAS performed to date analyze a relatively small number of strains (66). (ii) Since a key factor for successful genetic discovery is when strains that exhibit outlier responses are included in the analysis, the genetic analysis should not begin until after inbred strains that exhibit extreme DOA responses (i.e., top or bottom 10% and are >3-fold above (or below) the mean response of the other strains) have been identified. Preferably, the strain panel should include at least two strains that exhibit an extreme phenotypic response. Other investigators have used one or more of the various recombinant inbred (RI) strain panels for genetic mapping studies, which include: the Hybrid Mouse Diversity Panel (30 founder strains) (67, 68); the Diversity Outbred (69) and Collaborative Cross (70) panels (eight strains); and the BXD RI panel (71) (two strains). While these RI panels have proven to be useful for genetic mapping, they have a limitation. We do not know in advance which strains will exhibit outlier responses to current (or future) DOA that contribute to 21st century addiction-related public health problems, and the strains exhibiting outlier responses may not be among the founder strains for the existing RI panels. To use another disease as an example, Type 2 Diabetes Mellitus (T2DM), and its principal risk factor (obesity) have become a major 21st century public health problem (72); but the TallyHo strain is not among the founder strains used to construct any of the current RI panels. Nevertheless, TallyHo provides a valuable murine model for T2DM and obesity because it spontaneously develops hyperlipidemia, hyperglycemia, insulin resistance, and glucose intolerance (73, 74). A genetic analysis of diabetes—related traits among the inbred strains would miss important disease-causing genetic variants if the TallyHo strain was not included in the analysis.

While many different methods can be used to analyze GWAS data obtained from inbred stains, we have successfully used haplotype based computational genetic mapping (HBCGM) to identify murine genetic factors underlying 22 biomedical traits (5–9, 18, 20, 42, 64, 75–90). In an HBCGM experiment, a property of interest is measured in a panel of available mouse strains whose genomes have been sequenced; and then genetic factors are computationally predicted by identifying genomic regions (haplotype blocks) where the pattern of within-block genetic variation correlates with the distribution of the phenotypic responses among the strains (63, 64, 75) (Figure 1). However, a major barrier to genetic discovery is caused by the fact that HBCGM analyses generate many false positive associations, which appear along with the causative genomic region, for the trait response difference. This can make it difficult to identify the true causative genetic factor for a biomedical trait difference. Because of the ancestral relatedness of the inbred strains, some of the false positives are within genomic regions that are commonly inherited (a property referred to as “population structure”). Statistical methods have been developed to reduce the false discovery rate in GWAS studies by correcting for the population structure that exists that exists in humans (91, 92), plants (93), and mice (94). While these correction methods have substantial utility for analyzing human GWAS results, we have recently shown that population structure correction methods are less useful when analyzing murine GWAS results; and moreover, their use could increase the chance that a true causative genetic factor will be discarded (95). In brief, even though multiple genomic regions have a shared ancestral inheritance, one of them may be responsible for a phenotypic difference. To overcome this problem, we use filtering methods to identify the true causative factor from among the many correlated genomic regions. We have previously identified causative genetic factors from among the many genes with correlated allelic patterns by applying orthogonal criteria (64), which include gene expression, metabolomic (78), or curated biologic data (96), or by examining candidates within previously identified genomic regions (76, 77). This approach can provide results that are superior to that of a typical GWAS, which only uses a single highly stringent criterion to identify candidates. We recently analyzed 8,462 publicly available datasets of biomedical responses (1.52 M individual datapoints) measured in panels of inbred mouse strains. We found that our ability to identify the genetic basis for the biomedical trait differences among the inbred strains was enhanced when structured automated methods were used for filtering the genes output by HBCGM analyses (66). In that study, we selected correlated genes that were expressed in the target organ for the biomedical trait, had high impact SNP alleles, and where the published literature indicated that the gene had a functional relationship with the analyzed trait. Although we are in the early stage of using automated methods for assessing genetic results, we believe that the results from that study (66) provide an early indication of how “*augmented intelligence*” can be used to facilitate genetic discovery. For analysis of mouse genetic models for SUD, DOA-induced gene expression changes occurring in brain regions, which are known to be important sites for DOA responses (NAc, VTA, mPFC), can be analyzed to facilitate identification of causative genetic factors.

## Future Directions

We believe that genetic factors affecting DOA responses will be shared with those impacting learning and memory pathways (97). Multiple lines of evidence indicate that DOA “*hijack*” the neural circuits used for learning and memory (98–100). An organism's ability to learn and form memories is mediated by changes within neurons and brain circuits that are produced by changes in neuronal gene expression patterns, which are activated in response to stimuli (101). Synaptic plasticity, which are activity-based changes in synaptic transmission in neuronal networks, is a major component of learning and memory (102). Changes in presynaptic glutamate release as well as postsynaptic ionotropic glutamate receptor expression and subunit composition are associated with DOA-induced changes in neuroplasticity (103). Rapidly occurring changes in synaptic plasticity mediate DOA-induced behavioral effects, and they contribute to the acquisition of instrumental learning. By this mechanism, DOA-induced changes in synaptic plasticity can produce abnormally strong addiction-related memories. The effect of DOA on long-term potentiation (LTP) and long-term depression (LTD) has been well-studied in VTA dopaminergic neurons (104–106). For instance, cocaine exposure increases the AMPA/NMDA receptor ratio, alters GluA2-containing AMPARs, and decreases NMDA receptor functionality in VTA dopaminergic neurons (107–109). Structural plasticity, which is the formation of new synaptic boutons and dendritic spines, is also observed after DOA exposure (110). Increased dendritic spine density in the NAc and PFC are commonly observed changes in synaptic connections that contribute to the sequela of drug use (111–113). Circuit remodeling also occurs with DOA-induced dopamine-mediated responses. Specifically, DOA act on the mesolimbic dopaminergic pathway, which include the ventral tegmental area (VTA), nucleus accumbens (NAc) and associated limbic regions (114). The medial prefrontal cortex (mPFC), which exerts top-down excitatory glutamatergic control over the NAc and other downstream subcortical regions, might contribute to maladaptive behaviors (115). Different subregions of the mPFC (i.e., dorsal, and ventral infralimbic subregions) can both drive and inhibit drug seeking behaviors depending on the drug history and behavioral context (116). Dysfunction in these regions, such as hypoactivity or selective strengthening of the PFC-striatal pathway, could contribute to compulsion in drug addiction models (116, 117). Therefore, studying DOA-induced effects on synaptic and structural plasticity, as well as characterizing changes in neuronal circuitries, could greatly increase our understanding of DOA responses. Moreover, given the overlap between the neural circuits used for learning and those impacted by DOA, it is likely that there will be some degree of overlap between the genetic factors affecting responses to different types of abused drugs. Hence, it is also important to characterize the impact that genetic factors identified by analysis of mouse genetic models have on responses to different types of DOAs.

**Figure 2:**
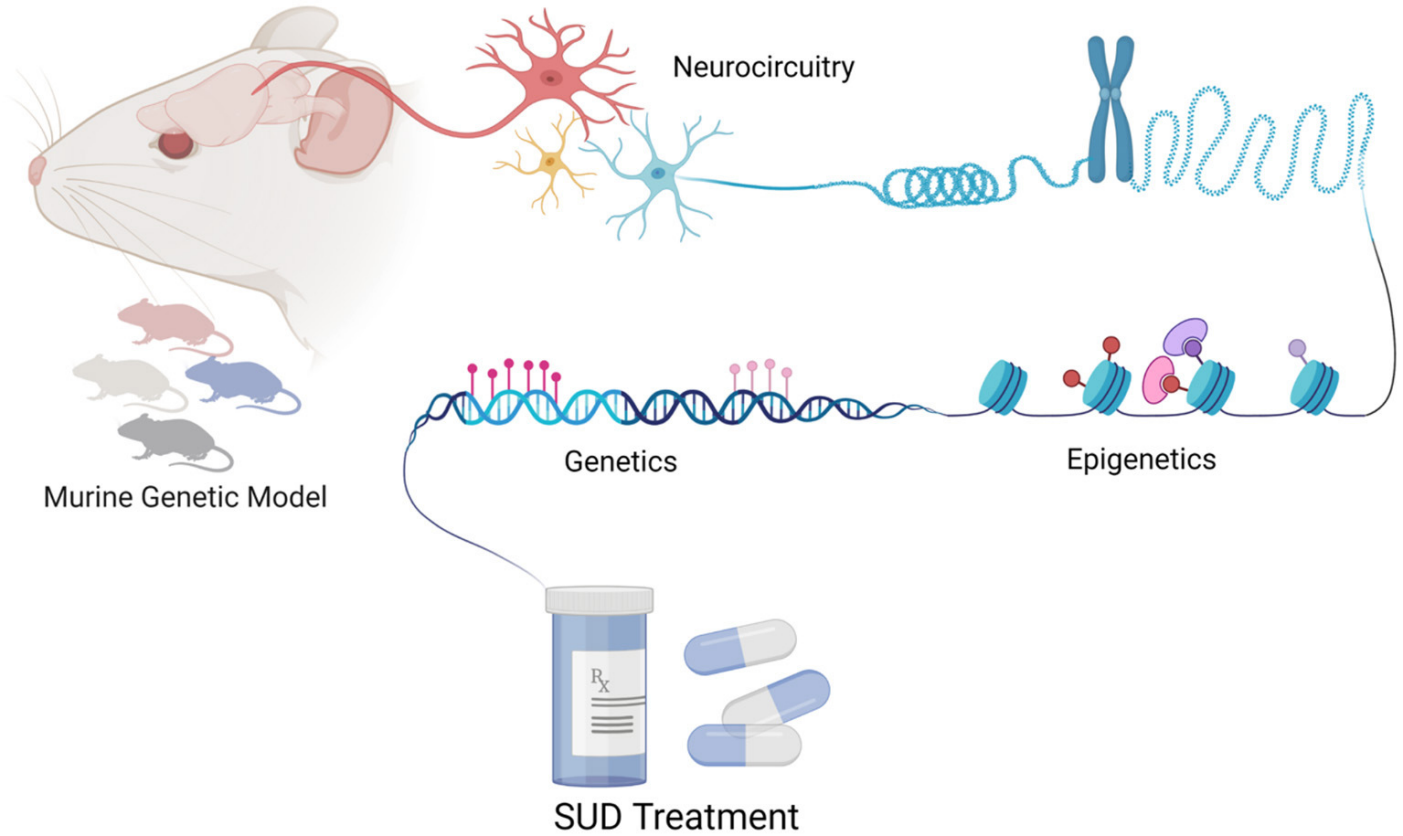
Image depicting how a mouse genetic model of a response related to a SUD can be analyzed to identify the genetic factors, epigenetic changes and the alterations in neurocircuits caused by a DOA. This diagram was created using BioRender.com software.

In addition to the transcriptional changes associated with neuronal plasticity, chromatin modifications are a major part of learning and memory processes (118–121). Much correlational evidence links changes in histones (predominantly acetylation) with short and long-term memory generation (121–123). Since the addiction state persists long after the period of DOA ingestion, DOA-induced epigenetic modifications are highly likely to be key contributors to addiction. Hence, DOA-induced chromatin structure changes in specific brain regions should be characterized along with DOA-induced transcriptional changes. The combined characterization of transcriptional and chromatin structure changes in the developing human brain has provided new insight into the mechanisms regulating brain development, and possibly into the pathobiology of psychiatric diseases (124). The methodology for simultaneously characterizing DOA-induced epigenetic and transcriptional changes in brain is now readily available (124). Characterization of DOA-induced chromatin structure changes in specific brain regions will provide the orthogonal information, which will facilitate the identification of genetic factors affecting addiction susceptibility. To do this, chromosomal regions with DOA-induced epigenetic changes can be examined to determine if they overlap with haplotype blocks that contain alleles that correlate with the pattern of DOA responses among the inbred strains. Also, linking genetic and epigenetic mechanisms with changes in synaptic circuit plasticity could lead to a deeper understanding of DOA-induced neuroadaptations (115) (Figure 2). For instance, DOA exposure produces region-specific epigenetic changes, which include an increase in global histone acetylation in the NAc, while this is reduced in the VTA (125, 126). Studying transcriptional and epigenetic changes in specific neuronal subpopulations is also important for understanding neural mechanisms and identifying novel therapeutic targets for prevention of addiction (127, 128). Thus, we believe that murine genetic models can be used to simultaneously characterize DOA-induced epigenetic and transcriptional changes, and for identifying genetic factors that alter DOA responses. Thus, murine models can provide the critically needed information that is required for successfully landing the airplanes whose flight path has been adversely affected by SUDs.

## Author Contributions

All authors listed have made a substantial, direct, and intellectual contribution to the work and approved it for publication.

## Funding

This work was supported by a NIH/NIDA award (5U01DA04439902) to GP.

## Conflict of Interest

The authors declare that the research was conducted in the absence of any commercial or financial relationships that could be construed as a potential conflict of interest.

## Publisher's Note

All claims expressed in this article are solely those of the authors and do not necessarily represent those of their affiliated organizations, or those of the publisher, the editors and the reviewers. Any product that may be evaluated in this article, or claim that may be made by its manufacturer, is not guaranteed or endorsed by the publisher.

## Abbreviations

CSA: cocaine self-administration
DOA: drugs of abuse
GWAS: genome wide association study
HBCGM: haplotype based computational genetic mapping
HIT: haloperidol induced toxicity
mCPP: morphine-induced conditioned place preference
NPOW: naloxone precipitated opiate withdrawal
NAc: nucleus accumbens
SUD: substance use disorder
VTA: ventral tegmental area.

## References

[1] Koob GF Ahmed SH Boutrel B Chen SA Kenny PJ Markou A. Neurobiological mechanisms in the transition from drug use to drug dependence. Neurosci Biobehav Rev. (2004) 27:739–49. 10.1016/j.neubiorev.2003.11.007 15019424

[2] Koob GF. Negative reinforcement in drug addiction: the darkness within. Curr Opin Neurobiol. (2013) 23:559–63. 10.1016/j.conb.2013.03.011 23628232

[3] Koob GF Volkow ND. Neurobiology of addiction: a neurocircuitry analysis. Lancet Psychiatry. (2016) 3:760–73. 10.1016/S2215-0366(16)00104-8 27475769 PMC6135092

[4] Peltz G Sudhof TC. The neurobiology of opioid addiction and the potential for prevention strategies. JAMA. (2018) 319:2071–2. 10.1001/jama.2018.3394 29710202

[5] Zheng M Zhang H Dill DL Clark JD Tu S Yablonovitch AL. The role of Abcb5 alleles in susceptibility to haloperidol-induced toxicity in mice and humans *PLoS Med*. (2015) 12:e1001782. 10.1371/journal.pmed.1001782 25647612 PMC4315575

[6] Zhang H Zheng M Wu M Xu D Nishimura T Nishimura Y. A Pharmacogenetic Discovery: Cystamine Protects against Haloperidol-Induced Toxicity and Ischemic Brain Injury. Genetics. (2016) 203:599–609. 10.1534/genetics.115.184648 26993135 PMC4858802

[7] Liang D Liao G Wang J Usuka J Guo YY Peltz G. A genetic analysis of opioid-induced hyperalgesia in mice *Anesthesiology*. (2006) 104:1054–62. 10.1097/00000542-200605000-00023 16645459 PMC1464476

[8] Liang DY Liao G Lighthall G Peltz G Clark JD. Genetic variants of the P-glycoprotein gene abcb1b modulate opioid-induced hyperalgesia, tolerance and dependence. Pharmacogenet Genomics. (2006) 16:825–35. 10.1097/01.fpc.0000236321.94271.f8 17047491

[9] Chu LF Liang D-Y Li X Sahbaie P D'arcy N Liao G. From mouse to man: the 5-HT3 receptor modulates physical dependence on opioid narcotics. Pharmacogenet. Genomics. (2009) 19, 193–205. 10.1097/FPC.0b013e328322e73d 19214139 PMC2730361

[10] Liang DY Li X Clark JD. 5-hydroxytryptamine type 3 receptor modulates opioid-induced hyperalgesia and tolerance in mice. Anesthesiology. (2011) 114:1180–9. 10.1097/ALN.0b013e31820efb19 21368652 PMC3085696

[11] Mckendrick G Graziane NM. Drug-induced conditioned place preference and its practical use in substance use disorder research. Front Behav Neurosci. (2020) 14:582147. 10.3389/fnbeh.2020.582147 33132862 PMC7550834

[12] Golden SA Jin M Shaham Y. Animal models of (or for) aggression reward, addiction, and relapse: behavior and circuits. J Neurosci. (2019) 39:3996–4008. 10.1523/JNEUROSCI.0151-19.2019 30833504 PMC6529864

[13] Reiner DJ Fredriksson I Lofaro OM Bossert JM Shaham Y. Relapse to opioid seeking in rat models: behavior, pharmacology and circuits. Neuropsychopharmacology. (2019) 44:465–77. 10.1038/s41386-018-0234-2 30293087 PMC6333846

[14] Farrell MR Schoch H Mahler SV. Modeling cocaine relapse in rodents: behavioral considerations and circuit mechanisms. Prog Neuropsychopharmacol Biol Psychiatry. (2018) 87:33–47. 10.1016/j.pnpbp.2018.01.002 29305936 PMC6034989

[15] Welsch L Bailly J Darcq E Kieffer BL. The negative affect of protracted opioid abstinence: progress and perspectives from rodent models. Biol Psychiatry. (2020) 87:54–63. 10.1016/j.biopsych.2019.07.027 31521334 PMC6898775

[16] Kest B Palmese CA Juni A Chesler EJ Mogil JS. Mapping of a quantitative trait locus for morphine withdrawal severity. Mamm Genome. (2004) 15:610–7. 10.1007/s00335-004-2367-3 15457340

[17] Kest B Hopkins E Palmese CA Adler M Mogil JS. Genetic variation in morphine analgesic tolerance: a survey of 11 inbred mouse strains. Pharmacol Biochem Behav. (2002) 73:821–8. 10.1016/S0091-3057(02)00908-5 12213527

[18] Liang DY Guo T Liao G Kingery WS Peltz G Clark JD. Chronic pain and genetic background interact and influence opioid analgesia, tolerance, and physical dependence. Pain. (2006) 121:232–40. 10.1016/j.pain.2005.12.026 16516386

[19] Bickel WK Stitzer ML Wazlavek BE Liebson IA. Naloxone-precipitated withdrawal in humans after acute morphine administration. NIDA Res Monogr. (1986) 67:349–54. 3092089

[20] Donaldson R Sun Y Liang D-Y Zheng M Sahbaie P Dill DL. The multiple PDZ domain protein Mpdz/MUPP1 regulates opioid tolerance and opioid-induced hyperalgesia. BMC Genomics. (2016) 17. 10.1186/s12864-016-2634-1 27129385 PMC4850636

[21] Bardo MT Rowlett JK Harris MJ. Conditioned place preference using opiate and stimulant drugs: a meta-analysis. Neurosci Biobehav Rev. (1995) 19:39–51. 10.1016/0149-7634(94)00021-R 7770196

[22] Tzschentke TM. Measuring reward with the conditioned place preference paradigm: a comprehensive review of drug effects, recent progress and new issues. Prog Neurobiol. (1998) 56:613–72. 10.1016/S0301-0082(98)00060-4 9871940

[23] Tzschentke TM. Measuring reward with the conditioned place preference (CPP) paradigm: update of the last decade. Addict Biol. (2007) 12:227–462. 10.1111/j.1369-1600.2007.00070.x 17678505

[24] Aguilar MA Rodriguez-Arias M Minarro J. Neurobiological mechanisms of the reinstatement of drug-conditioned place preference. Brain Res Rev. (2009) 59:253–77. 10.1016/j.brainresrev.2008.08.002 18762212

[25] Vanderschuren LJ Tjon GH Nestby P Mulder AH Schoffelmeer AN De Vries TJ. Morphine-induced long-term sensitization to the locomotor effects of morphine and amphetamine depends on the temporal pattern of the pretreatment regimen. Psychopharmacology (Berl). (1997) 131:115–22. 10.1007/s002130050273 9201798

[26] Vanderschuren LJ Schmidt ED De Vries TJ Van Moorsel CA Tilders FJ Schoffelmeer AN. A single exposure to amphetamine is sufficient to induce long-term behavioral, neuroendocrine, and neurochemical sensitization in rats. J Neurosci. (1999) 19:9579–86. 10.1523/JNEUROSCI.19-21-09579.1999 10531460 PMC6782918

[27] Kuhn BN Kalivas PW Bobadilla AC. Understanding addiction using animal models. Front Behav Neurosci. (2019) 13:262. 10.3389/fnbeh.2019.00262 31849622 PMC6895146

[28] Eisener-Dorman AF Grabowski-Boase L Tarantino LM. Cocaine locomotor activation, sensitization and place preference in six inbred strains of mice. Behav Brain Funct. (2011) 7:29. 10.1186/1744-9081-7-29 21806802 PMC3160884

[29] Wiltshire T Ervin RB Duan H Bogue MA Zamboni WC Cook S. Initial locomotor sensitivity to cocaine varies widely among inbred mouse strains. Genes Brain Behav. (2015) 14:271–80. 10.1111/gbb.12209 25727211 PMC4692246

[30] Cervantes MC Laughlin RE Jentsch JD. Cocaine self-administration behavior in inbred mouse lines segregating different capacities for inhibitory control. Psychopharmacology (Berl). (2013) 229:515–25. 10.1007/s00213-013-3135-4 23681162 PMC3770817

[31] Roberts AJ Casal L Huitron-Resendiz S Thompson T Tarantino LM. Intravenous cocaine self-administration in a panel of inbred mouse strains differing in acute locomotor sensitivity to cocaine. Psychopharmacology (Berl). (2018) 235:1179–89. 10.1007/s00213-018-4834-7 29423710 PMC5874174

[32] Bagley JR Khan AH Smith DJ Jentsch JD. Extreme phenotypic diversity in operant responding for an intravenous cocaine or saline infusion in the hybrid mouse diversity panel. BioRxiv [Preprint]. (2021). Available Online at: https://www.biorxiv.org/content/10.1101/2021.02.03.429584v1 10.1111/adb.13162 PMC9870574 35470554

[33] Bailey LS Bagley JR Dodd R Olson A Bolduc M Philip VM. Heritable variation in locomotion, reward sensitivity and impulsive behaviors in a genetically diverse inbred mouse panel. BioRxiv [Preprint]. (2021). 10.1111/gbb.12773 34672075 PMC9044817

[34] Weeks JR Collins RJ. Factors affecting voluntary morphine intake in self-maintained addicted rats. Psychopharmacologia. (1964) 6:267–79. 10.1007/BF00413156 5890552

[35] Collins RJ Weeks JR Cooper MM Good PI Russell RR. Prediction of abuse liability of drugs using IV self-administration by rats. Psychopharmacology (Berl). (1984) 82:6–13. 10.1007/BF00426372 6141585

[36] Woolverton WL. Determinants of cocaine self-administration by laboratory animals. Ciba Found Symp. (1992) 166:149–161; discussion 161–144. 10.1002/9780470514245.ch9 1638910

[37] Jentsch JD Taylor JR. Impulsivity resulting from frontostriatal dysfunction in drug abuse: implications for the control of behavior by reward-related stimuli. Psychopharmacology (Berl). (1999) 146:373–90. 10.1007/PL00005483 10550488

[38] de Wit H Phillips TJ. Do initial responses to drugs predict future use or abuse? Neurosci Biobehav Rev. (2012) 36:1565–76. 10.1016/j.neubiorev.2012.04.005 22542906 PMC3372699

[39] Piazza PV Deroche-Gamonet V. A multistep general theory of transition to addiction. Psychopharmacology (Berl). (2013) 229:387–413. 10.1007/s00213-013-3224-4 23963530 PMC3767888

[40] Richardson NR Roberts DC. Progressive ratio schedules in drug self-administration studies in rats: a method to evaluate reinforcing efficacy. J Neurosci Methods. (1996) 66:1–11. 10.1016/0165-0270(95)00153-0 8794935

[41] Venniro M Zhang M Caprioli D Hoots JK Golden SA Heins C. Volitional social interaction prevents drug addiction in rat models. Nat Neurosci. (2018) 21:1520–9. 10.1038/s41593-018-0246-6 30323276 PMC7386559

[42] Liang DY Zheng M Sun Y Sahbaie P Low SA Peltz G. The Netrin-1 receptor DCC is a regulator of maladaptive responses to chronic morphine administration. BMC Genomics. (2014) 15:345. 10.1186/1471-2164-15-345 24884839 PMC4038717

[43] Bradford D Cole SJ Cooper HM. Netrin-1: diversity in development. Int J Biochem Cell Biol. (2009) 41:487–93. 10.1016/j.biocel.2008.03.014 18455953

[44] Rajasekharan S Kennedy TE. The netrin protein family. Genome Biol. (2009) 10:239. 10.1186/gb-2009-10-9-239 19785719 PMC2768972

[45] Yetnikoff L Eng C Benning S Flores C. Netrin-1 receptor in the ventral tegmental area is required for sensitization to amphetamine. Eur J Neurosci. (2010) 31:1292–302. 10.1111/j.1460-9568.2010.07163.x 20345916

[46] Horn KE Glasgow SD Gobert D Bull SJ Luk T Girgis J. DCC expression by neurons regulates synaptic plasticity in the adult brain. Cell Rep. (2013) 3:173–85. 10.1016/j.celrep.2012.12.005 23291093

[47] Manitt C Mimee A Eng C Pokinko M Stroh T Cooper HM. The netrin receptor DCC is required in the pubertal organization of mesocortical dopamine circuitry. J Neurosci. (2011) 31:8381–94. 10.1523/JNEUROSCI.0606-11.2011 21653843 PMC6623341

[48] Goldman JS Ashour MA Magdesian MH Tritsch NX Harris SN Christofi N. Netrin-1 promotes excitatory synaptogenesis between cortical neurons by initiating synapse assembly. J Neurosci. (2013) 33:17278–89. 10.1523/JNEUROSCI.1085-13.2013 24174661 PMC6618363

[49] Sitek B Poschmann G Schmidtke K Ullmer C Maskri L Andriske M. Expression of MUPP1 protein in mouse brain. Brain Res. (2003) 970:178–87. 10.1016/S0006-8993(03)02338-2 12706259

[50] Shirley RL Walter NA Reilly MT Fehr C Buck KJ. Mpdz is a quantitative trait gene for drug withdrawal seizures. Nat Neurosci. (2004) 7:699–700. 10.1038/nn1271 15208631

[51] Karpyak VM Kim JH Biernacka JM Wieben ED Mrazek DA Black JL. Sequence variations of the human MPDZ gene and association with alcoholism in subjects with European ancestry. Alcohol Clin Exp Res. (2009) 33:712–21. 10.1111/j.1530-0277.2008.00888.x 19175764 PMC2819379

[52] Ehlers CL Walter NA Dick DM Buck KJ Crabbe JC. A comparison of selected quantitative trait loci associated with alcohol use phenotypes in humans and mouse models. Addict Biol. (2010) 15:185–99. 10.1111/j.1369-1600.2009.00195.x 20148779 PMC2848508

[53] Krapivinsky G Medina I Krapivinsky L Gapon S Clapham DE. SynGAP-MUPP1-CaMKII synaptic complexes regulate p38 MAP kinase activity and NMDA receptor-dependent synaptic AMPA receptor potentiation. Neuron. (2004) 43:563–74. 10.1016/j.neuron.2004.08.003 15312654

[54] Rama S Krapivinsky G Clapham DE Medina I. The MUPP1-SynGAPalpha protein complex does not mediate activity-induced LTP. Mol Cell Neurosci. (2008) 38:183–8. 10.1016/j.mcn.2008.02.007 18417361 PMC4057800

[55] Miyamoto Y Yamada K Nagai T Mori H Mishina M Furukawa H. Behavioural adaptations to addictive drugs in mice lacking the NMDA receptor epsilon1 subunit. Eur J Neurosci. (2004) 19:151–8. 10.1111/j.1460-9568.2004.03086.x 14750973

[56] Inturrisi CE. The role of N-methyl-D-aspartate (NMDA) receptors in pain and morphine tolerance. Minerva Anestesiol. (2005) 71:401–3. 16012411

[57] Ko SW Wu LJ Shum F Quan J Zhuo M. Cingulate NMDA NR2B receptors contribute to morphine-induced analgesic tolerance. Mol Brain. (2008) 1:2. 10.1186/1756-6606-1-2 18803856 PMC2546399

[58] Liaw WJ Zhu XG Yaster M Johns RA Gauda EB Tao YX. Distinct expression of synaptic NR2A and NR2B in the central nervous system and impaired morphine tolerance and physical dependence in mice deficient in postsynaptic density-93 protein. Mol Pain. (2008) 4:45. 10.1186/1744-8069-4-45 18851757 PMC2576175

[59] Erlendson MJ D'arcy N Encisco EM Yu JJ Rincon-Cruz L Peltz G., et al. Palonosetron and hydroxyzine pre-treatment reduces the objective signs of experimentally-induced acute opioid withdrawal in humans: a double-blinded, randomized, placebo-controlled crossover study. Am J Drug Alcohol Abuse. (2017) 43:78–86. 10.1080/00952990.2016.1210614 27712113 PMC5728104

[60] Elkomy M Sultan P Carvalho B Peltz G Wu M Clavijo C. Ondansetron pharmacokinetics in pregnant women and neonates: towards a new treatment for neonatal abstinence syndrome. Clin Pharmacol Ther. (2015) 97:167–76. 10.1002/cpt.5 25670522 PMC4325425

[61] Maas U Kattner E Weingart-Jesse B Schafer A Obladen M. Infrequent neonatal opiate withdrawal following maternal methadone detoxification during pregnancy. J Perinat Med. (1990) 18:111–8. 10.1515/jpme.1990.18.2.111 2366131

[62] American Academy of Pediatrics Committee on Drugs. Neonatal drug withdrawal. Pediatrics. (1998) 101:1079–86. 10.1542/peds.101.6.1079 9614425

[63] Wang J Liao G Usuka J Peltz G. Computational genetics: from mouse to man? Trends in Genetics. (2005) 21:526–32. 10.1016/j.tig.2005.06.010 16009447

[64] Zheng M Dill D Peltz G. A better prognosis for genetic association studies in mice. Trends Genet. (2012) 28:62–9. 10.1016/j.tig.2011.10.006 22118772 PMC3268904

[65] Beck JA Lloyd S Hafezparast M Lennon-Pierce M Eppig JT Festing MF. Genealogies of mouse inbred strains. Nat Genet. (2000) 24:23–5. 10.1038/71641 10615122

[66] Arslan A Guan Y Chen X Donaldson R Zhu W Ford M. High throughput computational mouse genetic analysis. BioRxiv. (2020). 10.1101/2020.09.01.278465

[67] Tewhey R Bansal V Torkamani A Topol EJ Schork NJ. The importance of phase information for human genomics. Nat Rev Genet. (2011) 12:215–23. 10.1038/nrg2950 21301473 PMC3753045

[68] Ghazalpour A Rau CD Farber CR Bennett BJ Orozco LD Van Nas A. Hybrid mouse diversity panel: a panel of inbred mouse strains suitable for analysis of complex genetic traits. Mamm Genome. (2012) 23:680–92. 10.1007/s00335-012-9411-5 22892838 PMC3586763

[69] Chick JM Munger SC Simecek P Huttlin EL Choi K Gatti DM. Defining the consequences of genetic variation on a proteome-wide scale. Nature. (2016) 534:500–5. 10.1038/nature18270 27309819 PMC5292866

[70] Chesler EJ Miller DR Branstetter LR Galloway LD Jackson BL Philip VM. The collaborative cross at Oak ridge national laboratory: developing a powerful resource for systems genetics. Mamm Genome. (2008) 19:382–9. 10.1007/s00335-008-9135-8 18716833 PMC2745091

[71] Belknap JK Crabbe JC. Chromosome mapping of gene loci affecting morphine and amphetamine responses in BXD recombinant inbred mice. Ann N Y Acad Sci. (1992) 654:311–23. 10.1111/j.1749-6632.1992.tb25977.x 1632590

[72] Centers for Disease Control Prevention (2020). National Diabetes Statistics Report, 2020. Atlanta, GA (2020). Available online at: https://www.cdc.gov/diabetes/data/statistics-report/index.html?CDC_AA_refVal=https%3A%2F%2F (accessed August 28, 2020).

[73] Kim JH Saxton AM. The TALLYHO mouse as a model of human type 2 diabetes. Methods Mol Biol. (2012) 933:75–87. 10.1007/978-1-62703-068-7_6 22893402

[74] Kim JH Sen S Avery CS Simpson E Chandler P Nishina PM. Genetic analysis of a new mouse model for non-insulin-dependent diabetes. Genomics. (2001) 74:273–86. 10.1006/geno.2001.6569 11414755

[75] Liao G Wang J Guo J Allard J Chang J Nguyen A. *In Silico* genetics: identification of a novel functional element regulating H2-Ea gene expression. Science. (2004) 306:690–5. 10.1126/science.1100636 15499019

[76] Smith SB Marker CL Perry C Liao G Sotocinal SG Austin JS. Quantitative trait locus and computational mapping identifies Kcnj9 (GIRK3) as a candidate gene affecting analgesia from multiple drug classes. Pharmacogenet Genomics. (2008) 18:231–41. 10.1097/FPC.0b013e3282f55ab2 18300945

[77] LaCroix-Fralish ML Mo G Smith SB Sotocinal SG Ritchie JG Austin JS. The β3 Subunit of the Na+,K+-ATPase affects pain sensitivity. Pain. (2009) 144:294–302. 10.1016/j.pain.2009.04.028 19464798 PMC2744953

[78] Liu H-H Lu P Guo Y Farrell E Zhang X Zheng M. An integrative genomic analysis identifies bhmt2 as a diet-dependent genetic factor protecting against acetaminophen-induced liver toxicity *Genome Res*. (2010) 20:28–35. 10.1101/gr.097212.109 19923254 PMC2798828

[79] Liu HH Hu Y Zheng M Suhoski MM Engleman EG Dill DL. Cd14 SNPs regulate the innate immune response. Mol Immunol. (2012) 51:112–27. 22445606 10.1016/j.molimm.2012.02.112 PMC3341513

[80] Grupe A Germer S Usuka J Aud D Belknap JK Klein RF. *In silico* mapping of complex disease-related traits in mice. Science. (2001) 92: 1915–8. 10.1126/science.1058889 11397946

[81] Rozzo SJ Allard J Choubey D Vyse T Izui S Peltz G. Evidence for an interferon-inducible gene, Ifi202, in the susceptibility to systemic lupus. Immunity. (2001) 15:435–43. 10.1016/s1074-7613(01)00196-0 11567633

[82] Guo YY Weller PF Farrell E Cheung P Fitch B Clark D. *In silico* pharmacogenetics: warfarin metabolism. Nat Biotechnol. (2006) 24:531–6. 10.1038/nbt1195 16680137 PMC1459533

[83] Guo YY Liu P Zhang X Weller PMM Wang J Liao G. *In vitro* and *in silico* pharmacogenetic analysis in mice. Proc Natl Acad of Sci USA. (2007) 104:17735–40. 10.1073/pnas.0700724104 17978195 PMC2077071

[84] Zaas AK Liao G Chein J Usuka J Weinberg C Shore D. Plasminogen alleles influence susceptibility to invasive aspergillosis. PLoS genetic. (2008) 4:e1000101. 10.1371/journal.pgen.1000101 18566672 PMC2423485

[85] Tregoning JS Yamaguchi Y Wang B Mihm D Harker JA Bushell ESC. Genetic susceptibility to the delayed sequelae of RSV infection is MHC-dependent, but modified by other genetic loci. J Immunol. (2010) 185:5384–91. 10.4049/jimmunol.1001594 20921522

[86] Hu Y Liang D Li X Liu H-H Zhang X Zheng M. The role of IL-1 in wound biology part I: murine *in silico* and *in vitro* experimental analysis. Anesth Analg. (2010) 111: 1525–33. 10.1213/ANE.0b013e3181f5ef5a 20889942

[87] Hu Y Liang D Li X Liu H-H Zhang X Zheng M. The role of IL-1 in wound biology part II: *in vivo* and human translational studies. Anesth Analg. (2010) 111:1534–42. 10.1213/ANE.0b013e3181f691eb 20889944

[88] Peltz G Zaas AK Zheng M Solis NV Zhang MX Liu H-H. Next-generation computational genetic analysis: multiple complement alleles control survival after candida albicans infection. Infect Immun. (2011) 79:4472–9. 10.1128/IAI.05666-11 21875959 PMC3257944

[89] Sorge RE Trang T Dorfman R Smith SB Beggs S Ritchie J. Genetically determined P2X7 receptor pore formation regulates variability in chronic pain sensitivity. Nat Med. (2012) 18:595–9. 10.1038/nm.2710 22447075 PMC3350463

[90] Ren M Kazemian M Zheng M He J Li P Oh J. Transcription factor p73 regulates Th1 differentiation. Nat Commun. (2020) 11:1475. 10.1038/s41467-020-15172-5 32193462 PMC7081339

[91] Reich DE Goldstein DB. Detecting association in a case-control study while correcting for population stratification. Genet Epidemiol. (2001) 20:4–16. 11119293 10.1002/1098-2272(200101)20:1<4::AID-GEPI2>3.0.CO;2-T

[92] Yu J Pressoir G Briggs WH Vroh Bi I Yamasaki M Doebley JF. A unified mixed-model method for association mapping that accounts for multiple levels of relatedness. Nat Genet. (2006) 38:203–8. 10.1038/ng1702 16380716

[93] Zhao K Aranzana MJ Kim S Lister C Shindo C Tang C. An arabidopsis example of association mapping in structured samples. PLoS Genet. (2007) 3:e4. 10.1371/journal.pgen.0030004 17238287 PMC1779303

[94] Kang HM Zaitlen NA Wade CM Kirby A Heckerman D Daly MJ. Efficient control of population structure in model organism association mapping. Genetics. (2008) 178:1709–23. 10.1534/genetics.107.080101 18385116 PMC2278096

[95] Wang M Fang Z Yoo B Bejerano G Peltz G. The effect of population structure on murine genome-wide association studies. Front Genet. (2021) 12:745361. 10.3389/fgene.2021.745361 34589118 PMC8475632

[96] Zhang X Liu H-H Weller P Tao W Wang J Liao G. *In silico* and *in vitro* pharmacogenetics: aldehyde oxidase rapidly metabolizes a p38 kinase inhibitor. Pharmacogenomics J. (2011) 11:15–24. 10.1038/tpj.2010.8 20177421

[97] Nestler EJ. Cellular basis of memory for addiction. Dialogues Clin Neurosci. (2013) 15:431–43. 10.31887/DCNS.2013.15.4/enestler 24459410 PMC3898681

[98] Nestler EJ. Molecular basis of long-term plasticity underlying addiction. Nat Rev Neurosci. (2001) 2:119–28. 10.1038/35053570 11252991

[99] Nestler EJ. Is there a common molecular pathway for addiction? Nat Neurosci. (2005) 8:1445–9. 10.1038/nn1578 16251986

[100] Russo SJ Dietz DM Dumitriu D Morrison JH Malenka RC Nestler EJ. The addicted synapse: mechanisms of synaptic and structural plasticity in nucleus accumbens. Trends Neurosci. (2010) 33:267–76. 10.1016/j.tins.2010.02.002 20207024 PMC2891948

[101] Guzman-Karlsson MC Meadows JP Gavin CF Hablitz JJ Sweatt JD. Transcriptional and epigenetic regulation of Hebbian and non-Hebbian plasticity. Neuropharmacology. (2014) 80:3–17. 10.1016/j.neuropharm.2014.01.001 24418102 PMC3984596

[102] Citri A Malenka RC. Synaptic plasticity: multiple forms, functions, and mechanisms. Neuropsychopharmacology. (2008) 33:18–41. 10.1038/sj.npp.1301559 17728696

[103] Volkow ND Michaelides M Baler R. the neuroscience of drug reward and addiction. Physiol Rev. (2019) 99:2115–40. 10.1152/physrev.00014.2018 31507244 PMC6890985

[104] Thomas MJ Malenka RC Bonci A. Modulation of long-term depression by dopamine in the mesolimbic system. J Neurosci. (2000) 20:5581–6. 10.1523/JNEUROSCI.20-15-05581.2000 10908594 PMC6772537

[105] Jones S Bonci A. Synaptic plasticity and drug addiction. Curr Opin Pharmacol. (2005) 5:20–5. 10.1016/j.coph.2004.08.011 15661621

[106] Liu QS Pu L Poo MM. Repeated cocaine exposure *in vivo* facilitates LTP induction in midbrain dopamine neurons. Nature. (2005) 437:1027–31. 10.1038/nature04050 16222299 PMC1457101

[107] Bellone C Luscher C. Cocaine triggered AMPA receptor redistribution is reversed in vivo by mGluR-dependent long-term depression. Nat Neurosci. (2006) 9:636–41. 10.1038/nn1682 16582902

[108] Mameli M Balland B Lujan R Luscher C. Rapid synthesis and synaptic insertion of GluR2 for mGluR-LTD in the ventral tegmental area. Science. (2007) 317:530–3. 10.1126/science.1142365 17656725

[109] Cheron J Kerchove d'Exaerde A. Drug addiction: from bench to bedside. Transl Psychiatry. (2021) 11:424. 10.1038/s41398-021-01542-0 34385417 PMC8361217

[110] Solinas M Belujon P Fernagut PO Jaber M Thiriet N. Dopamine and addiction: what have we learned from 40 years of research. J Neural Transm (Vienna). (2019) 126:481–516. 10.1007/s00702-018-1957-2 30569209

[111] Robinson TE Kolb B. Structural plasticity associated with exposure to drugs of abuse. Neuropharmacology. (2004) 47(Suppl 1):33–46. 10.1016/j.neuropharm.2004.06.025 15464124

[112] Lee KW Kim Y Kim AM Helmin K Nairn AC Greengard P. Cocaine-induced dendritic spine formation in D1 and D2 dopamine receptor-containing medium spiny neurons in nucleus accumbens. Proc Natl Acad Sci U S A. (2006) 103:3399–404. 10.1073/pnas.0511244103 16492766 PMC1413917

[113] Dos Santos M Cahill EN Bo GD Vanhoutte P Caboche J Giros B. Cocaine increases dopaminergic connectivity in the nucleus accumbens. Brain Struct Funct. (2018) 223:913–23. 10.1007/s00429-017-1532-x 29027032

[114] Kauer JA Malenka RC. Synaptic plasticity and addiction. Nat Rev Neurosci. (2007) 8:844–58. 10.1038/nrn2234 17948030

[115] Nestler EJ Luscher C. The molecular basis of drug addiction: linking epigenetic to synaptic and circuit mechanisms. Neuron. (2019) 102:48–59. 10.1016/j.neuron.2019.01.016 30946825 PMC6587180

[116] Moorman DE James MH Mcglinchey EM Aston-Jones G. Differential roles of medial prefrontal subregions in the regulation of drug seeking. Brain Res. (2015) 1628:130–46. 10.1016/j.brainres.2014.12.024 25529632 PMC4472631

[117] Pascoli V Hiver A Van Zessen R Loureiro M Achargui R Harada M. Stochastic synaptic plasticity underlying compulsion in a model of addiction. Nature. (2018) 564:366–71. 10.1038/s41586-018-0789-4 30568192

[118] Rudenko A Dawlaty MM Seo J Cheng AW Meng J Le T. Tet1 is critical for neuronal activity-regulated gene expression and memory extinction. Neuron. (2013) 79:1109–22. 10.1016/j.neuron.2013.08.003 24050401 PMC4543319

[119] Sweatt JD. The emerging field of neuroepigenetics. Neuron. (2013) 80:624–32. 10.1016/j.neuron.2013.10.023 24183015 PMC3878295

[120] Zovkic IB Guzman-Karlsson MC Sweatt JD. Epigenetic regulation of memory formation and maintenance. Learn Mem. (2013) 20:61–74. 10.1101/lm.026575.112 23322554 PMC3549063

[121] Lopez-Atalaya JP Barco A. Can changes in histone acetylation contribute to memory formation? Trends Genet. (2014) 30:529–39. 10.1016/j.tig.2014.09.003 25269450

[122] Gupta S Kim SY Artis S Molfese DL Schumacher A Sweatt JD. Histone methylation regulates memory formation. J Neurosci. (2010) 30:3589–99. 10.1523/JNEUROSCI.3732-09.2010 20219993 PMC2859898

[123] Halder R Hennion M Vidal RO Shomroni O Rahman RU Rajput A. DNA methylation changes in plasticity genes accompany the formation and maintenance of memory. Nat Neurosci. (2016) 19:102–10. 10.1038/nn.4194 26656643

[124] Trevino AE Muller F Andersen J Sundaram L Kathiria A Shcherbina A. Chromatin and gene-regulatory dynamics of the developing human cerebral cortex at single-cell resolution. Cell. (2021) 184:5053–69.e5023. 10.1016/j.cell.2021.07.039 34390642

[125] Martin TA Jayanthi S Mccoy MT Brannock C Ladenheim B Garrett T. Methamphetamine causes differential alterations in gene expression and patterns of histone acetylation/hypoacetylation in the rat nucleus accumbens. PLoS ONE. (2012) 7:e34236. 10.1371/journal.pone.0034236 22470541 PMC3314616

[126] Arora DS Nimitvilai S Teppen TL Mcelvain MA Sakharkar AJ You C. Hyposensitivity to gamma-aminobutyric acid in the ventral tegmental area during alcohol withdrawal: reversal by histone deacetylase inhibitors. Neuropsychopharmacology. (2013) 38:1674–84. 10.1038/npp.2013.65 23474591 PMC3717553

[127] Mews P Calipari ES. Cross-talk between the epigenome and neural circuits in drug addiction. Prog Brain Res. (2017) 235:19–63. 10.1016/bs.pbr.2017.08.012 29054289 PMC6339819

[128] Salery M Trifilieff P Caboche J Vanhoutte P. From signaling molecules to circuits and behaviors: cell-type-specific adaptations to psychostimulant exposure in the striatum. Biol Psychiatry. (2020) 87:944–53. 10.1016/j.biopsych.2019.11.001 31928716

